# A vaccine for SARS-CoV-2: goals and promises

**DOI:** 10.1016/j.eclinm.2020.100494

**Published:** 2020-07-29

It is the end of July 2020 and the pandemic is still soaring around the world. Former hot spots like China and most of Europe seem to have successfully beaten the first wave of the virus whereas countries like Brazil and the USA are still in the midst or even just at the beginning.

It becomes clear that the virus revealed strengths, weaknesses and inequality of our societies and health-care systems around the world. Crippling minorities and those who are less privileged all throughout the world. Strength can be seen in the few success stories of countries that were able to reduce the spread and keep the death rates relatively low. Such approaches were led by science, public health efforts and strong health-care systems that were prepared and had essential resources when they were needed. The situation in the USA is unique as the USA has one of the strongest economies, is equipped with cutting-edge medical infrastructure, and has top medical institutions, but yet the pandemic seems out of control. Lack of leadership, arrogance, and denouncements of science and medical experts has brought the USA into even worse dire straits. There is no national plan to defeat the virus, but the US government rather focuses on defunding and controlling organisations like the Centers for Disease Control and Prevention (CDC) to paint a better picture of the situation.

Now the race is on for a vaccine. Several candidates are in early clinical trials, but three are leading the field. One vaccine is being developed by the Chinese company CanSino Biologics and the phase 2 human trial is now published in *The Lancet* in July 2020. 508 eligible participants were enrolled, and significant humoral and cellular immune responses were seen within 28 days in most of the recipients of one vaccination dose. The second vaccine is being developed by the US company Moderna and tested by the US National Institute of Allergy and Infectious Diseases (NIAID). First results of the phase 1 were published in the *New England Journal of Medicine* in July 2020 and next phases are now underway. Given two vaccinations 28 days apart in 45 enrolled patients, the vaccine induced an immune response in all patients as indicated by serum-neutralising activity and T-cell responses. The third vaccine and the most advanced one is the Oxford vaccine developed by researchers at the University of Oxford, UK. The researchers teamed up with the British pharmaceutical company AstraZeneca to lead manufacturing and distribution efforts. The results of the phase 1 are now published in *The Lancet* in July 2020 and the vaccine trial has already moved into phase 2b/3. In the phase 1/2 study, 1077 participants were enrolled and randomly assigned to receive either the vaccine or placebo; 10 were participants were selected in a non-blinded manner to receive a booster vaccination. T-cell responses peaked on day 14, and neutralising antibodies were induced in all participant (10/10) after a second vaccine dose.

Whilst the trials are moving through their phases and first data are looking promising, we are now asking how we will determine that the vaccine is effective, or effective enough. Early data from recovered patients has shown that even though neutralising antibodies can be found in the blood, in some cases these fade fast and even become undetectable three months after infection. Will it be good enough to just dampen the immune reaction, or should we aspire to full immunity? Short-cuts have already been taken, given the fast development and progress of the candidates. The USA has set up a national programme called “Operation Ward Speed” (OWS) to accelerate the development of a safe and effective vaccine with the goal to be made available to Americans by January, 2021. Traditionally, vaccine developments take years to ensure long-term safety, durable immune response and beneficial outcomes. As a sobering thought, after 40 years of vaccine development efforts we still do not have a vaccine against HIV, the virus that causes AIDS. OWS is led by the leading medical institutions in the USA: CDC, Food and Drug Administration, and the National Institutes of Health are on the front of these COVID-19 countermeasures.

Alongside this programme, the Johns Hopkins Center for Health Security published their recommendations on how to ensure public acceptance of a vaccine. To achieve herd immunity with an effective vaccine, 60% of the population need to agree to be vaccinated. During a time, when leading scientists and institutions are being sidelined and discredited by political leaders, public doubt in this enterprise is anticipated. We need independent leadership to guide this effort, with integrity to ensure safety and efficacy of the vaccine. Political interests and pressures cannot overtake the oversight of one of the most important medical developments of our recent times. The next worst thing that could happen is to deploy a vaccine that does not work, creating a false sense of security and putting millions of people's heath at risk.

Once a vaccine is identified, another big concern will be the logistics of production and distribution. Both the Oxford and the Moderna vaccine are receiving funds by the Coalition for Epidemic Preparedness Innovations, which was launched as a global coalition in 2017 to coordinate intergovernmental actions to deploy vaccines in order to prevent future epidemics. One can speculate if global efforts would be put in place to ensure fair and equal access to the vaccine. To prevent what we have already seen with the buyout of remdesivir by the USA, we will need to think now, how to avoid a monopoly of only the richest countries and people gaining access to the vaccine. A strategy to avoid bidding wars is essential, to come together as one unit and coordinate a fair roll-out of the vaccine globally. The call for unity cannot be emphasized enough; we are already seeing how nations turn against each other and try to steal information on the vaccine development efforts. We should have learned by now that the search for a vaccine needs to be global effort, just as the fight against the spread needs to be a global response. The virus will only be defeated once it is beaten everywhere.

*EClinicalMedicine*

